# Deep learning based on 68Ga-PSMA-11 PET/CT for predicting pathological upgrading in patients with prostate cancer

**DOI:** 10.3389/fonc.2023.1273414

**Published:** 2024-01-08

**Authors:** Shiming Zang, Cuiping Jiang, Lele Zhang, Jingjing Fu, Qingle Meng, Wenyu Wu, Guoqiang Shao, Hongbin Sun, Ruipeng Jia, Feng Wang

**Affiliations:** ^1^ Department of Nuclear Medicine, Nanjing First Hospital, Nanjing Medical University, Nanjing, China; ^2^ Department of Ultrasound, Nanjing First Hospital, Nanjing Medical University, Nanjing, China; ^3^ Department of Urology, Nanjing First Hospital, Nanjing Medical University, Nanjing, China

**Keywords:** 68Ga-PSMA, pathological upgrading, prostate cancer, deep learning, PET/CT

## Abstract

**Objectives:**

To explore the feasibility and importance of deep learning (DL) based on 68Ga-prostate-specific membrane antigen (PSMA)-11 PET/CT in predicting pathological upgrading from biopsy to radical prostatectomy (RP) in patients with prostate cancer (PCa).

**Methods:**

In this retrospective study, all patients underwent 68Ga-PSMA-11 PET/CT, transrectal ultrasound (TRUS)-guided systematic biopsy, and RP for PCa sequentially between January 2017 and December 2022. Two DL models (three-dimensional [3D] ResNet-18 and 3D DenseNet-121) based on 68Ga-PSMA-11 PET and support vector machine (SVM) models integrating clinical data with DL signature were constructed. The model performance was evaluated using area under the receiver operating characteristic curve (AUC), accuracy, sensitivity, and specificity.

**Results:**

Of 109 patients, 87 (44 upgrading, 43 non-upgrading) were included in the training set and 22 (11 upgrading, 11 non-upgrading) in the test set. The combined SVM model, incorporating clinical features and signature of 3D ResNet-18 model, demonstrated satisfactory prediction in the test set with an AUC value of 0.628 (95% confidence interval [CI]: 0.365, 0.891) and accuracy of 0.727 (95% CI: 0.498, 0.893).

**Conclusion:**

A DL method based on 68Ga-PSMA-11 PET may have a role in predicting pathological upgrading from biopsy to RP in patients with PCa.

## Introduction

Systematic biopsy guided by transrectal ultrasound (TRUS) has long been considered a standard diagnostic method for confirming prostate cancer (PCa) in patients with elevated prostate-specific antigen level and/or abnormal digital rectal examination (1). However, this method often misses clinically significant PCa, and some patients experience a pathological upgrade following radical prostatectomy (RP) (2–4). Considering the crucial influence accurate diagnosis has on treatment decisions and prognosis prediction (5, 6), there is a pressing need to develop reliable methods for predicting the pathological upgrading of PCa.

Prostate-specific membrane antigen (PSMA) is a highly specific prostatic epithelial cell transmembrane protein and is highly expressed in PCa cells (7, 8). The recent emergence of 68Ga-labeled PSMA inhibitors as promising agents for positron emission tomography/computed tomography (PET/CT) in patients with primary PCa has demonstrated diagnostic and staging capabilities that are superior to conventional imaging techniques (9–11). Moreover, these PET/CT images can be quantitatively analyzed using deep learning (DL), which involves automatically extracting complex and abstract information from medical images to achieve highly accurate detection or classification outcomes (12, 13). Previous studies have shown the potential feasibility of DL in PCa detection, risk assessment, and prognosis prediction (12–16). However, to the best of our knowledge, the predictive value of DL based on 68Ga-PSMA-11 PET/CT for assessing pathological upgrading in PCa has not been investigated.

Therefore, this study was aimed at exploring an efficient strategy with a PSMA PET/CT–based convolutional neural network (CNN) model for predicting pathological upgrading in patients with PCa.

## Methods

### Patients and study design

We enrolled patients who had undergone 68Ga-PSMA-11 PET/CT prior to RP at our department between January 2017 and December 2022. The exclusion criteria were as follows: (1) lack of TRUS-guided systematic biopsy; (2) International Society of Urological Pathology grade group (ISUP GG) 5 on biopsy; (3) previous treatment for PCa before RP. The patients were randomly divided into a training cohort (n = 87) and a validation cohort (n = 22) with a ratio of 8:2 (**Supplementary Figure 1**).

### Data acquisition and preprocessing

All of the patients underwent PET/CT using a dedicated PET/CT system (United Imaging, uMI780, China) at 60 ± 5 min after intravenous injection of 2–2.3 MBq/kg 68Ga-PSMA-11 synthesized as previously described (17). A nonenhanced CT scan (120 kV, mA modulation, pitch 0.988, slice thickness 3.0 mm, increment 1.5 mm) was obtained, followed by a whole-body PET scan (3 min/bed, field of view 60 cm) in 3D mode (matrix 256 × 256) from the vertex to the proximal legs. The datasets were fully corrected for random coincidences, scatter radiation, and attenuation. For PET image reconstruction, the ordered-subsets expectation maximization method was used. Attenuation corrections of the PET images were performed using data from the CT scans. PET/CT fusion was performed using a workstation (uWS-MI, United Imaging).

The volumes of interest (VOIs) for the prostate gland were accurately delineated and segmented slice by slice using 3D Slicer software (version: 4.1.1.0; www.slicer.org) by a highly experienced nuclear medicine radiologist (FW) with 20 years of expertise in prostate PET/CT. The radiologist, blinded to the clinical information, performed this task by carefully analyzing the PET images and factoring in the corresponding CT scan for accurate localization and segmentation of the VOIs.

### Construction of deep learning models

**Figure 1:**
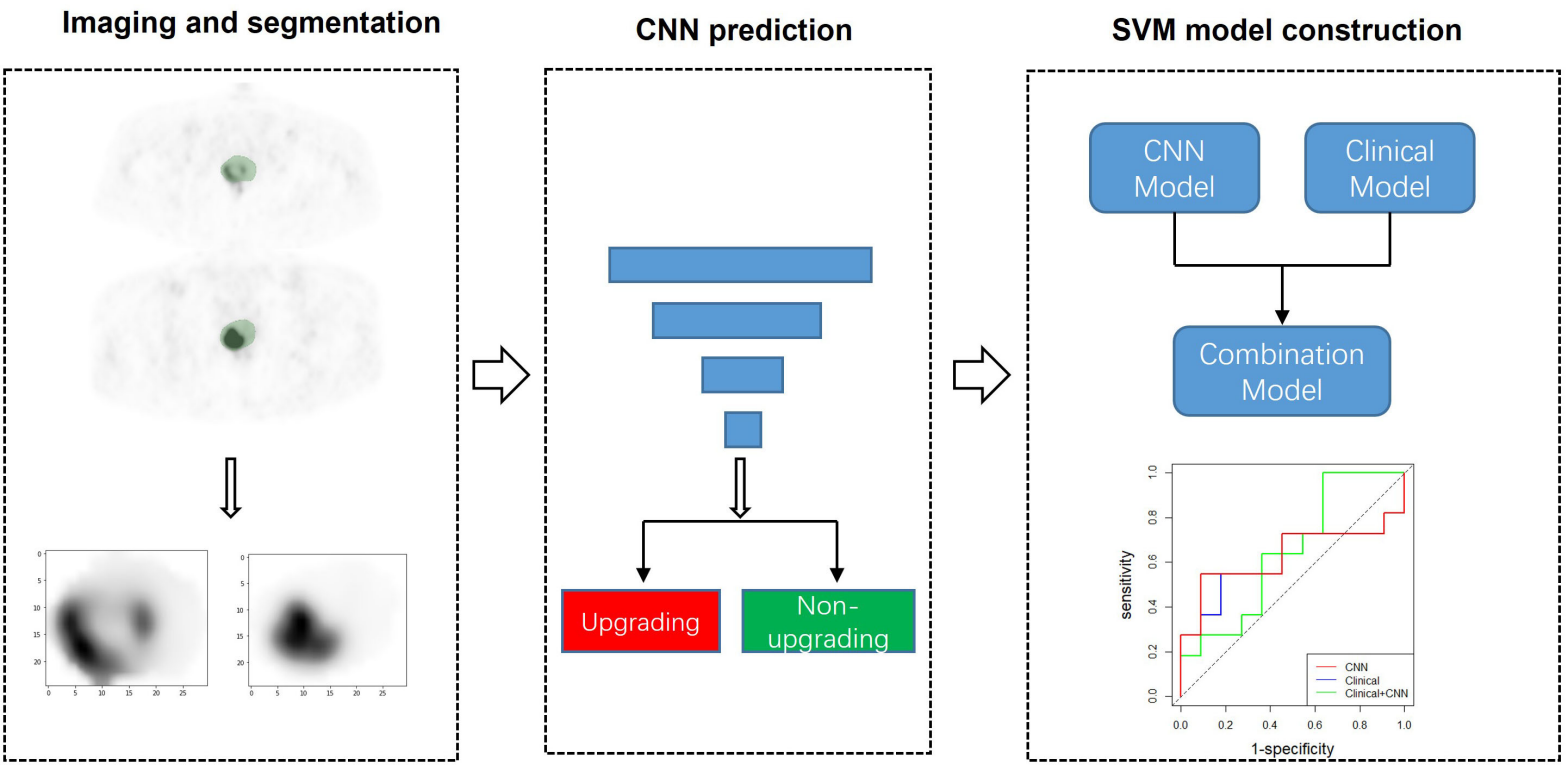
CNN modeling and analysis workflow. Using segmented 68Ga-PSMA-11 PET as input, two CNN models were trained to predict pathological upgrading. Additionally, the SVM models integrating clinical data with deep learning signature were constructed and evaluated. CNN, convolutional neural network; SVM, support vector machine.

The full flow diagram for machine learning models is provided in **Figure 1**. DL models were developed based on a convolutional neural network (CNN), which is a typical and commonly used DL architecture used to have been extensively applied for image analysis. CNN achieves prediction tasks by iterating the low-level information retrieved from input data into more abstract high-level features. Two well-known CNNs—ResNet-18 and DenseNet-121—were selected in this study (18, 19). To extract context features comprehensively, three-dimensional (3D) ResNet-18 and 3D DenseNet-121 were used to learn and extract the relevant image features from the PET images.

For model training, each VOI image was normalized according to the z-score by subtracting the means and then dividing by the standard deviations. To reduce overfitting, data augmentation of random rotation, horizontal/vertical-flip, and affine transformation were employed. The Adam optimizer was used to upgrade the network parameters with a batch size of 4. The learning rate was set at 0.0001. An early stopping criterion was used to terminate training as there was no further improvement in loss. All training sessions were implemented using PyTorch (version 1.12.1; https://www.pytorch.org). The performance of the developed model was evaluated with cross-validation (five-fold) on the training cohort and then tested on the test cohort.

### Construction of a clinical and combined model

The support vector machine (SVM) algorithm was used to evaluate the performance of clinical features for predicting pathology upgrading. GridsearchCV was carried out to select optimal hyperparameters for the SVM model, including kernel function, C, and sigma. Moreover, the signature [predicted possibilities of upgrading) of the CNN achieving higher AUC and clinical data (age, prostate-specific antigen (PSA), percentage of positive cores (PPC), biopsy ISUP GG, prostate volume, prostate-specific antigen density (PSAD)]] were jointly employed by the SVM to perform the multimodality prediction.

### Biopsy and pathological evaluation

All patients underwent 18-gauge needle biopsy under local anesthesia by experienced urologists. The biopsy was performed under the guidance of TRUS utilizing a 12-core extended scheme. The men subsequently underwent RP based on the joint decision between the treating surgeon and the patient, based on current clinical guidelines. Upgrading was defined as any increase of biopsy ISUP GG to RP ISUP GG.

### Statistical analysis

The Mann–Whitney U test was used to compare continuous variables, while the chi-squared test was used for the comparison of categorical variables. The diagnostic performance of the models for predicting pathological upgrading was evaluated using receiver operating characteristic (ROC) curve analysis. The optimal cutoff values of the models were determined by maximizing the Youden index in the training set. The fixed model cutoff values from the training set were then applied to the test set. Statistical analysis was conducted using SPSS 22.0 (IBM, Armonk, NY, USA) and R software (version 4.1.3; http://www.Rproject.org). All statistical tests were two-sided, and a P value < 0.05 was considered statistically significant.

## Results

### Clinical characteristics

In total, 109 patients were finally included in the study and divided randomly into the training set (n = 87) and the test set (n = 22). The baseline characteristics of the patients in the training and test sets are summarized in **Table 1**. In the training set, 50.57% of the patients (44 of 87) were identified as upgrading and 49.43% (43 of 87) as non-upgrading. In the test set, 50.00% of the patients (11 of 22) were identified as upgrading and 50.00% of the patients (11 of 22) as non-upgrading. Based on those characteristics of the patients, no significant differences were observed between the training and test sets.

**Table 1:** Clinical characteristics in the training and test sets.

Characteristic	Training set (n = 87)	Test set (n = 22)	*p* value
Age (years)	71.81 (68–76)	70.91 (66.25–75.25)	0.677
PSA (ng/mL)	20.54 (8.86–25.12)	18.88 (8.42–24.67)	0.970
Prostate volume (mL)	40.80 (26.53–47.94)	34.85 (22.95–41.90)	0.214
PSAD	0.54 (0.24–0.66)	0.68 (0.28–0.91)	0.110
PPC	45.05 (25.00–71.43)	37.01 (8.33–58.33)	0.264
Biopsy ISUP GG			0.986
1	29 (33.33)	7 (31.82)	
2	21 (24.14)	6 (27.27)	
3	30 (34.48)	7 (31.82)	
4	7 (8.05)	2 (9.09)	
Whole-gland ISUP GG			0.796
1	12 (13.79)	3 (13.64)	
2	19 (21.84)	5 (22.73)	
3	34 (39.08)	8 (36.36)	
4	18 (20.69)	5 (22.73)	
5	14 (16.09)	1 (4.55)	
Pathological outcomes			0.971
Upgrading	44 (50.57)	11 (50.00)	
Stable	40 (45.98)	10(45.45)	
Downgrade	3 (3.45)	1(4.55)	
T stage			0.869
2	57 (65.52)	14 (63.64)	
3	30 (34.48)	8 (36.36)	

Continuous variables are presented as median (interquartile range; IQR), while categorical variables are presented as patients (%).

PSA, prostate-specific antigen; PSAD, prostate-specific antigen density; PPC, percentage of positive cores; ISUP GG, International Society for Urological Pathology grade group.

### Performance of the deep learning models

The AUC, accuracy, sensitivity, and specificity of the two CNNs measured in the cross-validation are illustrated in **Supplementary Table 1** and **Supplementary Figure 2**. The overall performance of the 3D ResNet-18 model was slightly better than that of the 3D DenseNet-121 model (AUC: 0.621 vs. 0.559 [*p* = 0.443], accuracy: 0.644 vs. 0.609 [*p* = 0.638]). The 3D ResNet-18 model achieved sensitivity of 0.682 (95% confidence interval [CI]: 0.523, 0.809) and specificity of 0.605 (95% CI: 0.445, 0.706). Additionally, the signature of the 3D ResNet-18 model and clinical data were considered as input variables to build the combined model.

### Performance of the models in the test set

**Figure 2:**
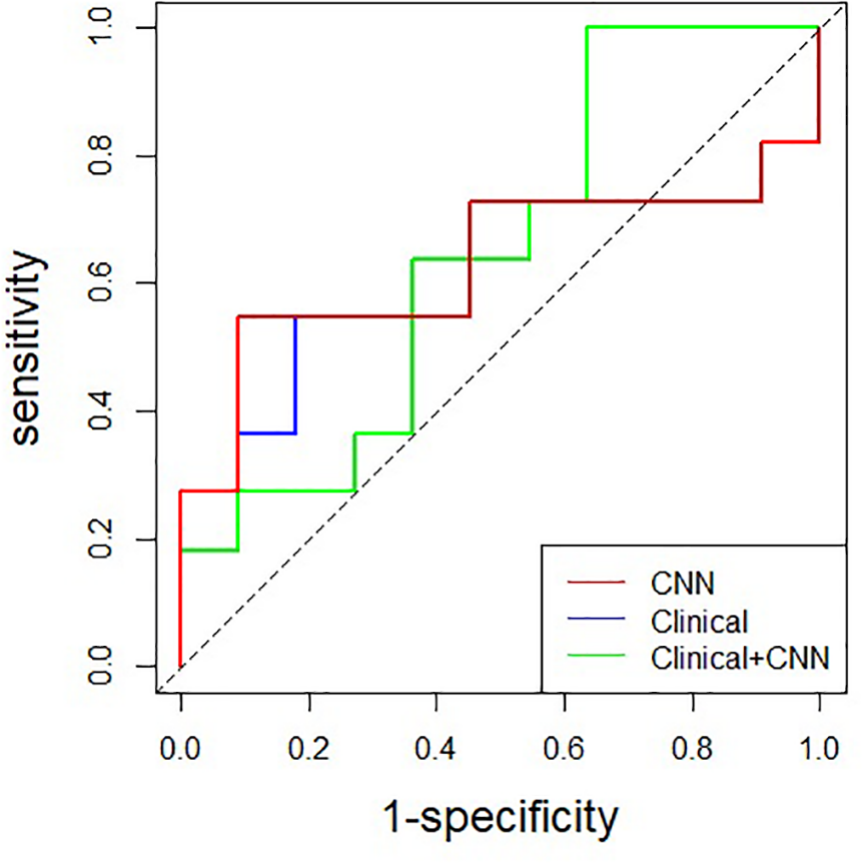
Comparison of receiver operating characteristic curves among CNN, clinical and combined model in the test set (AUC: 0.612, 0.645, and 0.628, respectively). CNN, convolutional neural network.

The performance of the CNN only, clinical data only, and combined model is summarized in **Table 2** and **Figure 2**. The 3D ResNet-18 model yielded an AUC value of 0.612 (95% CI: 0.350, 0.873) and accuracy of 0.682 (95% CI: 0.546, 0.828), which was comparable to the clinical model (*p* = 0.863 and *p* = 1.000, respectively). The 3D ResNet-18 model performed comparable on specificity to the clinical model (0.818 vs. 0.364, *p* = 0.080), but worse on sensitivity (0.546 vs. 1.000, *p* = 0.035). The obtained CNN performance in the test set was comparable to cross-validation (*p* = 0.949).

**Table 2:** Prediction performance of the CNN, clinical, and combined model in the test set.

Model	AUC (95% CI)	Accuracy (95% CI)	Sensitivity (95% CI)	Specificity (95% CI)
CNN	0.612 (0.350, 0.873)	0.682 (0.546, 0.828)	0.546 (0.246, 0.819)	0.818 (0.478, 0.968)
Clinical	0.645 (0.402, 0.887)	0.682 (0.451, 0.861)	1.000 (0.679, 1.000)	0.364 (0.124, 0.684)
Clinical + CNN	0.628 (0.365, 0.891)	0.727 (0.498, 0.893)	0.546 (0.246, 0.819)	0.909 (0.571, 0.995)
Comparisons among the CNN, clinical, and combined model (*p* values)				
CNN vs. clinical	0.863	1.000	0.035	0.080
CNN vs. CNN+clinical	0.406	0.741	1.000	1.000
Clinical vs. CNN+clinical	0.930	0.741	0.012	0.024

CNN, convolutional neural network; AUC, area under the curve; CI, confidence interval.

For multimodality analysis, the combined model achieved the highest accuracy (0.727 [95% CI: 0.498, 0.893]) among the models, although there were no significant differences in AUC (0.628 vs. 0.612, *p* = 0.406), accuracy (0.727 vs. 0.682, *p* = 0.741), sensitivity (0.546 vs. 0.546, *p* = 1.000), and specificity (0.909 vs. 0.818, *p* = 1.000) between the combined model and the 3D ResNet-18 model. No significant differences were found in AUC (0.628 vs. 0.645, *p* = 0.930) and accuracy (0.727 vs. 0.682, *p* = 0.741) when comparing the combined model with the clinical model. However, the combined model demonstrated lower sensitivity (0.546 vs. 1.000, *p* = 0.012) and higher specificity (0.909 vs. 0.364, *p* = 0.024) than the clinical model.

## Discussion

Histopathology results of biopsy samples and the biopsy Gleason score of the patients play a key role in clinical decision-making such as in choosing RP, extended lymphadenectomy, radiotherapy, or active surveillance (20–22). The present study demonstrated that the CNNs trained using PSMA PET/CT data performed well in predicting pathological upgrading from TRUS-guided systematic biopsy to final RP, which has never been investigated before. The 3D ResNet-18 model was successfully validated in the independent test set (AUC, 0.612; accuracy, 0.682; sensitivity, 0.546; specificity, 0.818).The combined model achieved significantly higher specificity but lower sensitivity than the clinical model.

Pathological upgrading from biopsy to RP is common in clinical practice. Serefoglu et al. (4) assessed 90 patients and reported an upgrading rate of 47.8% in those who underwent TRUS-guided systematic biopsy. Another study documented an upgrading rate of 31.5% in patients who had undergone the same procedure (23). On account of precise targeting, multiparametric magnetic resonance imaging targeted biopsy significantly improved the detection of clinically significant PCa and decreased the detection of insignificant PCa. In our study, we observed upgrading rates of 50.57% and 50.00% in the training and test sets, respectively. The variation in upgrading rates may be due to the exclusion of all patients with ISUP GG 5 at biopsy. Moreover, previous studies recorded different upgrading rates in patients with different risk groups or biopsy cores (24, 25). Considering that an adverse pathological outcome can potentially lead to erroneous decisions, the development of an efficient predictive model has great significance.

In recent years, there have been many studies assessing DL techniques based on PSMA PET/CT for detection and risk stratification of PCa. Zhao et al. (15) found that a CNN was able to accurately detect the bone and lymph node lesions on 68Ga-PSMA-11 PET/CT. Capobianco et al. (16) constructed a CNN model trained on 68Ga-PSMA PET/CT data to both classify PET/CT regions of interest as uptake suspicious or nonsuspicious for cancer and assign them an anatomical location classification. The evaluated algorithm showed satisfactory agreement with expert assessment for identification and anatomical location classification of suspicious uptake sites. In this study, we focused on predicting pathological upgrading and developed a CNN model based on 3D ResNet-18 net architecture for the prediction. The satisfactory performance of the CNN model suggests that DL has great potential for predicting pathological upgrading.

Previous studies have investigated the use of PSMA PET/CT in predicting pathological upgrading (26, 27). However, most of these studies focused on evaluating the standardized uptake value (SUV) of the prostate lesion, without considering the extensive spatial and morphological information available. Our study showcased the ability of 3D CNN models to extract comprehensive insights from the entire prostate to predict pathological upgrading. In order to eliminate the influence of surrounding organ tissues, the VOI of the prostate was segmented as the input volume for the CNN models. Moreover, we integrated clinical features with CNN to create a multimodality model so as to comprehensively predict pathological upgrading. Although the overall performance was satisfactory, no significant differences were found in AUC and accuracy when comparing the combined model with the CNN model, which could be due to the small size of our sample and consequent overfitting.

This study has some limitations. First, its single-center design and relatively small sample size may compromise the model’s generalization ability and affect its sensitivity and specificity. Therefore, in order to improve the robustness of the model, it is necessary to formulate a unified standard for multicenter studies, and identify and test multicenter data using DL methods. Second, further research using different PET/CT scanners is needed to validate the generalizability and robustness of the CNN model. Third, different observers who performed segmentation could have influenced the stability of the model. Automated and accurate tumor segmentation must be developed to facilitate the efficiency of the DL process.

## Conclusion

The effective CNN model based on 68Ga-PSMA PET/CT may have a role in predicting pathological upgrading from biopsy to RP in patients with primary PCa.

## Data availability statement

The original contributions presented in the study are included in the article/**Supplementary Material**. Further inquiries can be directed to the corresponding authors.

## Ethics statement

The studies involving humans were approved by the Ethic Committee of Nanjing First Hospital. The studies were conducted in accordance with the local legislation and institutional requirements. The participants provided their written informed consent to participate in this study.

## Author contributions

SZ: Software, Writing – original draft, Conceptualization. CJ: Data curation, Methodology, Writing – original draft. LZ: Data curation, Methodology, Writing – original draft. JF: Formal Analysis, Validation, Writing – original draft. QM: Software, Writing – original draft. WW: Methodology, Writing – original draft. GS: Resources, Writing – original draft. HS: Resources, Writing – review & editing. RJ: Writing – review & editing. FW: Conceptualization, Writing – review & editing.
