# Supplementary material for: Deep learning based on 68Ga-PSMA-11 PET/CT for predicting pathological upgrading in patients with prostate cancer
Source: Front Oncol. 2024 Jan 8;13:1273414. doi: 10.3389/fonc.2023.1273414 (PMC10800856; doi:10.3389/fonc.2023.1273414)
Supplement: Supplementary file 1 [file DataSheet_1.docx]

Supplementary Material

Table S1 Prediction performance of the two CNN models

| Model | AUC (95% CI) | *P* value | Accuracy (95% CI) | *P* value | Sensitivity (95% CI) | Specificity (95% CI) |
| --- | --- | --- | --- | --- | --- | --- |
| ResNet | 0.621 (0.500, 0.742) | 0.443 | 0.644 (0.534, 0.744) | 0.638 | 0.682 (0.523, 0.809) | 0.605 (0.445, 0.746) |
| DenseNet | 0.559 (0.436, 0.683) |  | 0.609 (0.499, 0.712) |  | 0.273 (0.155, 0.430) | 0.953 (0.829, 0.992) |

CNN, convolutional neural network; AUC, area under the curve; CI, confidence interval


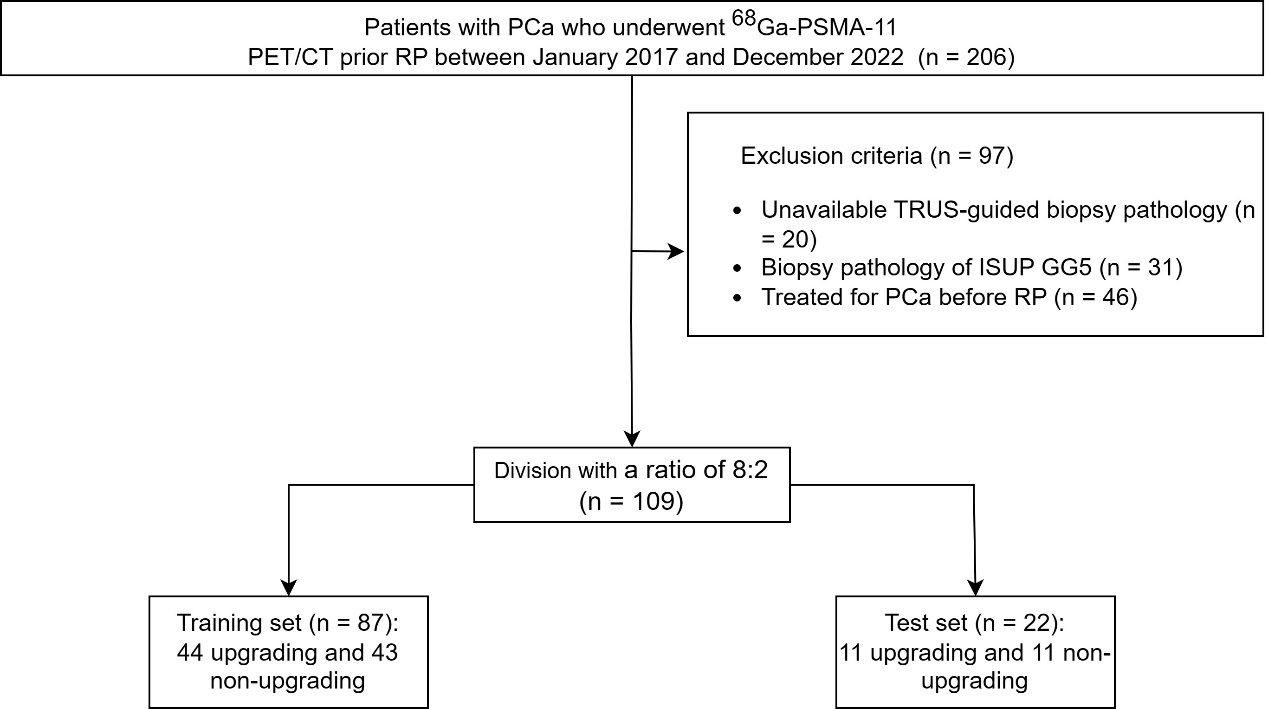


**Figure S1** Flowchart of the study. PCa, prostate cancer; RP, radical prostatectomy; TRUS, transrectal ultrasound; ISUP GG, International Society for Urological Pathology grade group.


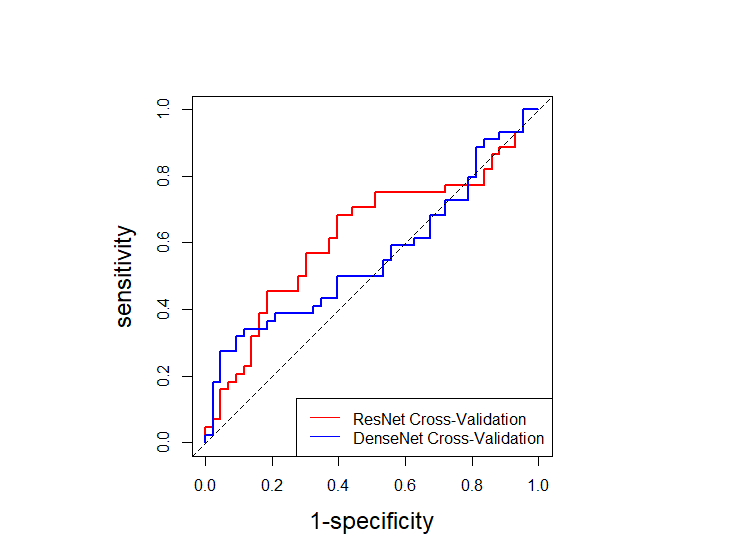


**Figure S2** Prediction performance of the two CNN models in cross-validation.
